# Presence of the Micrococcus in the Blood of Malignant Measles; Its Importance in Treatment

**Published:** 1882-08

**Authors:** John M. Keating

**Affiliations:** Lecturer on Diseases of Children in the University of Pennsylvania, Visiting Obstetrician to the Philadelphia Hospital


THE PRESENCE OF THE MICROCOCCUS IN THE BLOOD OF MALIGNANT MEASLES; ITS IMPORTANCE IN TREATMENT. By JOHN M. KEATING, M.D., Lecturer on Diseases of Children in the University of Pennsylvania, Visiting Obstetrician to the Philadelphia Hospital. Read June 7, 1882.

I propose to present for your consideration this evening the report of a recent epidemic in the Children's Asylum of the Philadelphia Hospital. The ward in which the disease first showed itself contained children between the ages of two and three years; some of them had been deserted by their mothers, and others had been placed there temporarily while the mothers were employed in dutics about the establishment. For the most part, these children presented a fair appearance of health; they were seemingly well nourished, of good development, though probably they would have been classed as "strumous," if their large features and tendency to glandular enlargements and eczematous eruptions had received careful attention. Together with all children of this class living in asylums, they certainly presented an open field for the production of those complications that are usually such fatal attendants upon measles.

In order to save time, I shall in this paper simply narrate the history of the cases. The little patients were zealously cared for by Dr. H. E. Campbell, resident physician, to whom I am indebted for the record of the notes taken at each of my visits.

I shall also embody in this report the investigation undertaken by Dr. Henry F. Formad, now well-known as the patient and thorough investigator of the microscopic appearance of the blood in diphtheria, associated with Dr. H. C. Wood, under the auspices of the National Board of Health. Dr. Formad examined almost daily the blood of each little patient, and together we noted the presence of micrococci in the malignant cases, and their absence in those of mild type; a record of these examinations was kept by Dr. W. A. Edwards, assistant pathologist, as also the records of the post-mortem examinations, and to him I am indebted for them. We entered this study with no pre-conceived views, the rapidity with which this exceedingly fatal epidemic came upon us, necessitated careful study in order to attempt, if possible, to discover its cause. I present also some photographs which show well the appearance of the field in these cases.

Case I.—J. F., aged two years and three months, was taken sick April 12, 1882. There had been no cases of well-defined measles in the house at that time, although it was epidemic in the city. The child had a sore throat, some cough, with fever. The throat eruption was punctated and well marked. The child died in convulsions April 15, 1882.

*Autopsy.*—An ante-mortem (chicken fat) clot was found in the right heart, extending into the pulmonary artery. There was great systemic venous engorgement. A decided staining was noticed upon the cadaver, especially about the temple, which caused the remark that the disease was probably one of the exanthemata. This child had been placed upon carbonate of ammonia, quinia, and digitalis; had had bromide of potassium, hot baths, and stimulants in small quantities.

Case II.—M. J., aged three years and five months, was taken sick April 9, 1882. A slight eruption on the face was noted April 11, 1882; the next day the eruption was complete over the entire body. In this case the eruption was irregular, was decidedly that of measles, but of malignant type. Still she did well until April 17, 1882, when suddenly the breathing became harsh, and pulse was rapid. At 4.30 P.M., had an attack of suffocation, with rapid feeble pulse. There was general venous engorgement, the face and lips were purple; pulse growing more rapid. Hot baths, with mustard and frictions, were used, and carbonate of ammonia and digitalis given. It was suggested to bleed in this case, but the evidences of heart clot were so marked, and the child had exhibited so many evidences of malignancy at the onset, that baths were used instead. April 11, 1882, at 3

A.M. she was still growing worse despite treatment, and shortly after had a violent convulsion and died.

There was no autopsy permitted.

Case III.—A. G., æt. 16 months. Took sick April 15, 1882.

April 16th had prodromes of measles. The temperature and pulse ran as follows:

16th.— P. M., 102°.
17th.—A. M., 101°; P. M., 103°; pulse, 180.
18th.—A. M., 102°; P. M., 103°; pulse, 180.
19th.—A. M., 101°; P. M., 102°; pulse, 160.
20th.—A. M., 101°; P. M., 102°; pulse, 148.
21st.—A. M., 100°; P. M., 100°; pulse, 180.

In this case no defined eruption appeared on the body, although there was a decided papular eruption on the uvula and anterior half arches of the throat. Whilst showing this case to the class we noted streaks of grayish membrane in the fauces. The child had decided bronchitis, and the voice showed that the laryngeal mucous membrane was also affected. This child was not at that time very ill. There was no question in our minds at the time that it was affected severely by the measles poison, that it was, in fact, another malignant case. It was carefully watched, the nourishment regularly given with quinia and iron daily, as was customary in all the cases.

On the 21st the breathing was noted as peculiar (I shall describe it hereafter). An emetic was ordered of ipecac, fearing an accumulation of mucus from the bronchitis present, and also carbonate of ammonia and digitalis and hot foot baths were given. The child's intelligence seemed good, and it will be noted that the temperature was but 100°, while the pulse was 180.

At 8 P. M. a second attack of suffocation occurred, and the child died in violent convulsions. The venous engorgement was very marked.

*Autopsy.*—A. G., baby, æt. one year. Post-mortem examination held ten hours after death.

*Heart.* Left side and valves all normal.

*Right side.* A large ante-mortem clot filling the cavity of the right ventricle, and extending into the auricle; a clot was also seen in the pulmonary artery.

*Lungs.* Left, normal.

*Right lung.* At the base of this lung the lesions of pulmonary congestion were seen, especially where the lung approximates the diaphragm.

*Intestines.* Slightly congested and hyperæmic.

*Mesenteric glands.* Enlarged and infiltrated by simple congestion.

*Kidneys.* Normal.

*Blood.* Taken from the heart cavity as soon as it was opened, and examined, showed micrococci in the liquor sanguinis and in the white blood corpuscles.

CASE IV.—J. F. McH., æt. 23 months. This child had a typical attack of measles. The case was shown to my ward class several times throughout its course, the eruption was studied carefully in all of its details, and my friend, Dr. John M. Taylor, obtained for me an excellent representation of the measles eruption in water-colors from this case. I refer especially to these points as evidence that the case was one of measles; the cases heretofore were so irregular as to leave room for doubt to those hearing the recital of their histories. The eruption was rapidly disappearing, and desquamation had set in. April 21, the bronchitis seemed to be aggravated, the respirations were 36, and expirations seemed unusually prolonged. The breathing was noisy; the heart's action was rapid; pulse 148. Suddenly, in the evening, an attack of suffocation came on, which was relieved by an inhalation of nitrite of amyl. On the morning of April 22 the heart was beating 168; the venous engorgement was very marked, the jugular veins standing out like whip cords; the respirations were from 36 to 40, but the temperature was 99°. I saw the child at this time, and noted the gasping breathing, the feeble pulse, and the tumultuous action of the heart. There seemed to be capillary spasm, judging from the gasping breath, the imploring look which the child gave to all its attendants, and we at once gave an inhalation of nitrite of amyl. In a few moments it seemed relieved. The administration of carbonate of ammonia, digitalis, hot baths, etc., was rigidly adhered to.

The child seemed comfortable until 4:30 P. M., when it had another attack of milder character, though longer duration, and in it, finally died of convulsions. It was observed, says Dr. Campbell in his notes, that the convulsion was not as severe as in the previous cases.

*Autopsy.*—J. F. McH., post-mortem made twenty hours after death.

Eruption not well-marked.

*Heart.* Right ventricle contained a small ante-mortem clot. This clot was in the cavity of the ventricle, and did not involve the valves, either tricuspid or pulmonary. The left side of the heart was normal in every respect; contained no clot.

*Lungs.* Normal, with the exception of hypostatic congestion at both bases. The pulmonary and costal pleura of the left side were inflamed, and adherent in some places.

*Trachea.* Inflamed, and containing a tenacious mucous secretion.

*Larynx.* Inflamed and hyperæmic.

*Liver.* Normal.

*Intestines.* Peyer's patches and the solitary and agminated glands infiltrated and hyphræmic.

*Mesenteric glands.* Enlarged and infiltrated; they were about the size of a grain of corn.

*Kidneys.* Normal.

*Spleen.* Amyloid bodies enlarged until they presented almost the appearance seen in a tubercular spleen.

*Blood.* Taken from heart as soon as punctured. Micrococci were found in the liquor sanguinis and in the white blood corpuscles, and they were mobile. In the corpuscles they were seen in great numbers in active movement, of a vibratory or whirling character, and they appeared to have devoured the white cells. No bacilli were seen.

CASE V.—J. McG., æt. 26 months. Ordinary case of measles. The eruption had disappeared on or before April 15.

April 22. Child restless; marked bronchitis; cough paroxysmal upon waking, especially after excitement; mucous râles coarse, and fine throughout lungs posteriorly; throat congested, and saliva at times tinged with blood. At this date the breathing was noted as noisy. Pulse 144; respirations 32; temperature 100°.

April 23. Mucous râles becoming general, and not limited to areas as heretofore; pulse 152; respirations 34; temperature 101. In addition to the tonic and stimulating treatment, mustard poultices were applied to thorax, and hot baths frequently given. Carbonate of ammonia was given now, gr. ij, every hour, and bisulphate of quinia by suppository, gr. ij, every three hours. In addition to this, the child was given, as were all the others, milk and lime-water, beef-tea, etc., at frequent intervals. We also used in this case the frequent administration of small doses of syr. ipecac, to relieve the secretion, which was abundant and tenacious.

April 23. Evening. Pulse was rapid; 160 to 170; temperature $102\frac{1}{2}°$; breathing becoming labored and gasping (fish-like); venous stasis was becoming more marked. Increased the whisky to about one ounce a day. The attacks of suffocation continued paroxysmally; the jugular veins stood out like cords at times. Nitrite of amyl gave immediate relief, but relapse soon followed; it was always followed by free emesis, which seemed to be in itself beneficial. About midnight a severe paroxysm came on, and with it a convulsion in which the child died. After death the venous engorgement was more marked, and heart clot had been suspected for some time before. This little patient was the first case whose blood Dr. Formad examined during life; the view of the fluid was photographed; micrococci were found in great abundance, acting especially on the white corpuscles. The blood was examined very shortly before the child's death, when the symptoms of heart clot had been fairly established, and the case declared hopeless.

Unfortunately, no autopsy was permitted in this case.

CASE VI.—F. M., æt. $2\frac{1}{2}$ years. This case ran a course as did the others, and I will only occupy time with a description of the post-mortem appearances.

*Autopsy.*—F. M., æt. $2\frac{1}{2}$ years. Eruption well-marked on mucous membrane of buccal cavity; not so on cutaneous surface.

Upon laying thorax open, lungs found to be anæmic, as far as arterial circulation was concerned, but dammed up with venous blood.

*Heart.* Normal in size and weight.

*Right side* contained a clot extending along the pulmonary artery for some distance ; it was chicken fat in consistence.

*Left Side.* Normal.

*Spleen.* Congested; weight four ounces.

*Intestines.* Along the small intestines could be seen a few Peyer's patches inflamed, and well outlined against the comparatively normal gut. The mesenteric glands presented a very good example of enlargement and infiltration; they looked like so many peas scattered throughout the mesentery.

*Liver.* Normal.

*Kidneys.* Normal.

*Brain.* Not examined.

*Blood.* Taken from heart cavity as soon as it was open showed micrococci in the liquor sanguinis and in the white blood corpuscles, in abundance; they were not mobile. A number of zoöglæa masses were seen.

Case VII.—C. M., aged two and a half years. The eruption in the throat of this child was very well marked. A few crescentic points appeared in the temples, and the case rapidly developed malignant symptoms.

April 21. Slight grayish suspicious patches of membrane are seated in the throat. The child is hoarse, and there is much bronchitis.

April 22. Pulse rapid; respiration, 28; breathing irregular. There is great general venous stasis, the skin dark and mottled.

Dr. Formad examined the blood microscopically and found it full of micrococci. He took a specimen sample for photography. Prognosis very unfavorable, as the child has fluttering heart and gasping breathing. Hot baths had been used freely with no success, so also salicylic acid, which had been suggested early in the disease.

After consultation with Dr. Formad, the account of which I incorporate in the summary, it was concluded to give at once ℥ij of whisky, and repeat it every hour; milk was continued as the only other food.

April 24. Pulse, 144; temperature, 101°; respiration, 48. Circulation much improved. Venous engorgement relieved; breathing greatly improved. The child continued to improve during the day. At the end of the twenty-four hours it had taken six ounces of whisky, and yet it showed no effects of alcoholism. At noon the pulse was 140, respiration, 36; 6 P. M. pulse 132, respiration, 32; 11 P. M., pulse 132, respiration, 26, and regular, breathing easy, though somewhat noisy, but not harsh.

April 25, A. M., temperature 98°, pulse 128, respiration 26; P. M., temperature 98°, pulse 108, respiration, 24.

April 26, A. M., temperature 98°, pulse 96, respiration 22; P. M., temperature 98°, pulse 104, respiration, 24.

The respirations remained regular, and the child continued to improve.

After the examination of the blood on April 30, owing to the relative increase of the blood corpuscles, it was decided to give Fowler's solution of arsenic gtt. ij, three times daily. The large doses of whisky were kept up for three or four days, and gradually diminished.

I give Dr. Formad's reports, which he kindly wrote out for me:

Microscopic examination of the blood in the above case (examination made with a one-sixteenth immersion lens).

Examination April 22, 1882.—Blood full of micrococci (sphero-bacteria), affecting many of the white blood corpuscles; also a large quantity of these fungi free and in various forms of grouping, mostly in zoöglæa masses. White blood corpuscles are in increased quantity; precipitation of fibrine excessively marked under the glass.

April 24. (Same case.) Micrococci present, but in diminished quantity; white blood corpuscles less affected; precipitation of fibrine less marked.

April 26. (Same case.) Micrococci very marked, yet principally in zoöglæa masses and free in serum, but not affecting the white corpuscles, although the latter are in increased quantity; fibrine not noticeable.

April 30. (Same case.) Micrococci present; white blood corpuscles still in excess, but not affected by micrococci. Red blood corpuscles not readily forming rouleaux, having lost partly their bi-concavity.

May 3. (Same case.) Micrococci present in diminished quantity. White blood corpuscles diminishing in quantity.

May 7. (Same case.) Same as last.

May 18. (Same case.) Still some few micrococci present; blood otherwise appears normal.

CASE VIII.—J. W., æt. 6 months. May 13, P. M. Eruption appeared on fifth day. The temperature ran as follows:

May 13.—A. M., P. M. 100 3-5°.
May 14.—A. M., 101 2-5°; P. M. 102 3-5.
May 15.—A. M., 101; P. M., 103 2-5.
May 16.—A. M., 101 2-5; P. M. 104.
May 17.——A. M., 101; P. M. 102 3-5.
May 18.—A. M., 102; P. M. 103 2-5.
May 19.—A. M., 100; P. M. 103.
May 20.—A. M., 103 2-5; P. M. 102.
May 21.—A. M., 105; death.

May 13. A fever mixture was given during the day. Quinia et ferri citratis, gr. ij, every three hours.

May 18. The eruption fading, but leaving a purple stain and mottled appearance of skin. Catarrhal pneumonia or collapse probably exists, as the bronchitis is very extensive, the râles numerous, and subcrepitant. The blood examined under the microscope shows micrococci in the blood corpuscles, but none free in the field. They are seen in great numbers.

May 19, P. M. For the past two hours the child has been very restless; the breathing rapid and labored, and also spasmodic. No membrane on tonsils or fauces. Heart's action very rapid; venous stasis marked especially in the jugular veins. Gave hot baths (says Dr. Campbell's notes), and covered him with blankets, with some relief. Increased the whisky to ʒij every hour.

In the evening gave an emetic. The child at night was breathing easier; friction sounds heard.

May 20. At times the strangulation would seem imminent.

The venous engorgement increased, and the child died in convulsions on the morning of the 21st.

The post-mortem examination showed pneumo niaand pleurisy, with effusion.

The following eight cases were all taken sick at once, and I shall simply give a general statement of them for the purpose of especially calling attention to the case of W. L.

J. J., æt. four years; W. L., æt. five years; E. C., æt. three years, catarrhal bronchitis; W. W., æt. three years; C. B., æt. two years, catarrhal bronchitis; J. W., æt. five years; J. D., æt. five years; L. K., æt. five years

Of these eight, seven presented severe, but nevertheless typical examples of measles, and their blood was carefully examined by Dr. Formad, and found normal. The case of W. L., who was taken ill at the same time as the others, showed from the onset a malignant tendency, giving a record such as I have already described. Dr. Formad gave me the following as the result of the examination of the blood in this case, and I had frequent occasion of examining it with him myself. Let me say, that as soon as the presence of micrococci was established, the child was placed upon ʒij doses of whisky every hour. Quiniæ et ferri citras in citric acid, gr. ij, every three hours, friction to the extremities, and warm baths, with milk and beef tea.

April 22. A few micrococci seen in the field.

April 26. Again noted.

April 30. Micrococci still present, white corpuscles increased, and marked precipitation of fibrine. None were noted as having penetrated the corpuscles; those that were found were simply in the serum. This child recovered, though every indication gave a very unfavorable prognosis.

In presenting this detailed report, I desire to call especial attention to the following points, viz.: the microscopic examination of the blood and the constant association of micrococci with the general manifestations of malignancy (a condition already well known), and the gradual but positive amelioration of all bad symptoms by treatment, which was directed to the micrococci as the *fons et origo* of trouble (this, I believe, for the first time exhibited).

It will be noted that the post-mortem examination of these cases showed more or less simple pulmonary congestion, and at times simple enlargement of the glands, but usually so circumscribed as to preclude the possibility of its being the immediate, or even remote cause of death. Again, the mode of death was peculiar: the fatal signs came on suddenly and with frightful intensity; the gasping breathing; the frantic efforts to obtain air (or really to aerate the blood); the imploring look, with consciousness not impaired, seemingly unduly acute, until the final convulsion or gradual cyanosis brought the end. The turgid veins, the occasional venous engorgement, the feeble pulse, and the fluttering heart, pointed unmistakably to but one cause; the gradually forming right-sided heart-clot; and the post-mortem appearance, as these notes show, gave us a large, tough, chicken-fat clot, obstructing the venous circulation, firmly planted in the right heart and its tributaries, which was too often exhibited to raise a question. One of the earliest symptoms of this impending danger was undue rapidity of respiration. The child seemed to be doing well; its eruption irregular, probably incomplete, or dark and mottled, and in blotches, when attention would be called to the great rapidity of respiration, with a peculiar gasping inspiration, fish-like in character. The other fatal symptoms would follow rapidly, and within twelve hours the child, despite carbonate of ammonia, warm baths, digitalis, etc., would die of heart-clot. What caused this?

In a short paper which appeared in the *American Journal of Medical Sciences* for January, 1882, I gave the experience of a number of cases of diphtheria, scarlet fever and measles, and then attributed the condition to an increase of fibrine due to the rapid tissue changes and the malignancy of the type of disease, and urged the importance of pushing an alkaline treatment from the start.

The microscope has shown here that something more is associated with this condition.

The moment that symptoms of malignancy, viz dark eruptions, ill-defined crescents, delayed and imperfect appearance of the eruption, with feeble circulation, high temperature, and pharyngeal false membrane appear, the examination of the blood showed micrococci in abundance in the field. They do not simply lie as impediments to the free passage of blood, though they most undoubtedly do this, and obstruct its passage in capillaries, but they surround the corpuscles, they enter the white corpuscles, and there develop with surprising rapidity, and finally cause some of them to rupture, and their contents will cover the field. Still, if they alone clogged the circulation in the capillaries, caused stasis in the lung, and thereby provoked an accumulation in the already enfeebled right heart, with blood having a tendency to coagulate, the cause of heart-clot alone would seem explained.

We find that they develop with activity, when the blood current is retarded, hence we find them spread throughout the heart-clot itself, possibly at times having been here arrested by the obstruction to the flow caused by the lung congestion, known as a frequent complication of these cases, and finally aiding, by a mechanical cause alone, the deposition of fibrine that forms the clot. They do more. They act upon the white blood corpuscle, destroy it in all probability, or, at least, as one of the cases proves conclusively, prevent its change to red corpuscles, and thus the oxygen carriers being either destroyed or reduced in numbers with none to replace them, the tissues retain their detritus for want of carriers to relieve them, and another factor is added to increase mortality.

Granted, then, that the appearance of micrococci is coincident with symptoms of malignancy, we must assert that, whether their association be *post hoc* or *propter hoc*, they must have common cause ; our treatment receives an impetus in a new direction.

I asked Dr. Formad what, in his experience, most readily checked the developement of micrococci in his culture solutions obtained from erysipelas, diphtheria, etc., he answered, alcohol. Dr. Campbell at once withdrew carbonate of ammonia and digitalis from the treatment for the future, and gave whisky. Five children had already died with the symptoms I have just described, and the sixth was exhibiting all the malignant symptoms, together with those which experience had taught us came from commencing heart-clot. The child had rapid gasping breathing, was becoming cyanosed, its heart was tumultuous, and the rapid pulse was growing weaker. The instructions were to give three ounces of whisky within the next twelve hours, in frequent and small doses. The treatment was carefully carried out, and the child was saved. In this child micrococci were found in abundance in the blood, but none had penetrated the corpuscles, and for a long time the preponderance of white blood corpuscles was noted, which continued until gradually the blood became normal under the use of arsenic.

Again, let me illustrate another point. In one ward there were six cases at the height of eruption. I carefully examined, with Drs. Campbell and Markoe, each case. One case was found to be of a malignant type. The child's right cheek was hardened and inflamed, and the mucous membrane showed that glistening surface so manifest in cancrum oris. The breath was fetid, there were cerebral symptoms, and a grayish exudation lined the fauces. We wished to test with the microscope, so without reference to any particular case, we requested Dr. Formad to examine the blood of all. In five the blood showed no micrococci, in one a large mass appeared in the field upon the first examination, and this one was the malignant case. This child was placed at once upon large doses of whisky, and it was also given, in tonic doses, quiniæ et ferri citras and citric acid.

The vegetable acids have also this remarkable effect of checking the devolopment of micrococci in culture solutions, especially acetic acid, but the mineral acids, also carbolic acid, it is said, have no such action.

The bichloride of mercury also possesses this quality to a very marked degree.

Now let me, for a moment, review this supject in the light of treatment, which to us is certainly of greatest importance. We may look at present upon the micrococcus as associated with the malignant symptoms of all complications known as "blood-poisoning." It is found in erysipelas, in puerperal septicæmia, in diphtheria, and in malignant measles. Experience has already taught us that alcohol, the vegetable acids, calomel or corrosive sublimate, are the drugs *per se* in septicæmia.

The action of alcohol and calomel is too well authenticated in puerperal septicæmia to doubt their efficacy.

We know of late how surprising a result will often attend the use of alcohol and corrosive sublimate in malignant diphtheria, and also the value of vegetable acids, especially lemon-juice and claret, in this dreaded disease.

My cases simply illustrate one part of the subject. In this recital I do not allude to the other death-producing complications which are so universal. Children with measles will die of cerebral complications of pneumonia, of enteritis, and entero colitis—with these we have nothing to do at present. Their treatment will, of course, depend upon lesions—quinine, opium, hot baths, poultices will all take part.

I have simply brought forward the subject of " blood-poisoning for your consideration, and as these remarks are based upon the careful study of but one epidemic, they cannot be submitted as conclusive, but simply as illustrative of what may, at some future time, be accomplished by studying, not merely the bacteria anatomically and physiologically, but by experimentation with bactericides as antidotal in their action, in diseases, they may cause or complicate.

The conclusions which seem warranted by the statements of this paper, and by observations made in other cases in the Hospital, are as follows :—

The micrococcus is found in the contents of pustules and vesicles, and also in the blood taken from measles-papules in ordinarily mild cases, without its being present in the blood taken from the punctured finger. In severe cases, called malignant in this paper, owing to the rapid appearance of morbid symptoms, the blood shows early in the attack numerous patches of micrococcus in the field.

In cases of rapid sthenic disease with high temperature and great tissue change, the evidences of large quantities of fibrine with a tendency to coagulation are manifest. The rapid production of micrococci soon gives the mechanical impediment, and if stasis takes place from any other obstruction to the circulation, clots rapidly form.

The non-appearance of clots in malignant fevers attended with fluid blood, such as low forms of typhus, diphtheria, etc, is simply due to the fact that rapid tissue changes have resulted in decomposition, instead of into fibrine-forming substances—no fibrine is formed, hence no clots—but the micrococci are present all the same. These cases are held by some to be the malignant ones, think the *foudroyante* character of the others, just mentioned, entitle them to be placed in the same category.

But the micrococcus, if left unheeded, may attack the white corpuscle, as distinctly seen under the microscope, and destroy its contents. The red cells also change in appearance, and finally probably become, to all intents and purposes, useless in the economy. When such a condition is seen by the microscope and found extensive, a fatal prognosis can be given, despite the most active treatment.

In cases where the white blood cells are as yet unaffected, treatment, when active, will be followed by good results, provided the other complications, as visceral inflammation, etc, are not in themselves excessive.

Alcohol (whisky in our cases) seems in some way, when given in large amounts, to check the progress of the marauders, to arrest the process of destruction, and, if needful, can be associated with quinine and iron in small repeated doses, digitalis perhaps, and frictions, baths and poultices, etc. As we have seen, the symptoms presented are contemporary with the changes going on within the blood; they may, in lieu of a careful microscopic examination of the blood, be taken as a gauge for treatment; knowing what can and will take place, early active treatment will give the patient some chance for the future.

---

WE have to express our indebtedness to the author of the preceding article, Dr. J. M. Keating, of Philadelphia, for sending us advance sheets of his exceedingly interesting paper. The author neglected to iuform us of the name of the journal if any, in which his paper is to appear, and it is for that reason only here omitted. Should we be in position to credit it to the proper source in another issue, we shall be glad to perform this proper duty.

